# Determinants of resilience to cigarette smoking among young Australians at risk: an exploratory study

**DOI:** 10.1186/1617-9625-8-7

**Published:** 2010-07-08

**Authors:** Yola Colgan, Deborah A Turnbull, Antonina A Mikocka-Walus, Paul Delfabbro

**Affiliations:** 1 School of Psychology, University of Adelaide, Level 4, Hughes Building, Adelaide 5005, SA, Australia; 2 School of Nursing and Midwifery, University of South Australia, City East Campus, Adelaide 5001, SA, Australia

## Abstract

**Background:**

Numerous researchers studied risk factors associated with smoking uptake, however, few examined protective factors associated with smoking resilience. This study therefore aims to explore determinants of smoking resilience among young people from lower socioeconomic backgrounds who are at risk of smoking.

**Methods:**

Overall, 92 out of 92 vocational education students accepted invitation to participate in this exploratory study. The Adelaide Technical and Further Education (TAFE) Arts campus was chosen for the study given the focus on studying resilience in young people of lower socioeconomic status i.e. resilient despite the odds. A self-report questionnaire comprising a measure of resilience: sense of coherence, sense of humour, coping styles, depression, anxiety and stress, and family, peers and community support, was distributed among participants aged 15 to 29. Additional factors researched are parental approval and disapproval, course type, and reasons for not smoking. Using the Statistical Package for the Social Sciences (SPSS, version 13.0), analyses were undertaken using frequencies, means, standard deviations, independent sample t-tests, correlations, analysis of variance, logistic regression, and chi-square test.

**Results:**

Twenty five (27%) out of 92 students smoked. Young people with peer support tended to smoke (p < .05). A relationship between daily smoking and depression, anxiety and stress was also found (p < .05). When both mothers and fathers disapproved of their children smoking, it had a greater influence on females not smoking, compared with males. The majority of students chose 'health and fitness' as a reason for not smoking. Students in the Dance course tended to not smoke.

**Conclusions:**

The current study showed that most students chose 'health and fitness' as the reason for not smoking. Single anti-smoking messages cannot be generalised to all young people, but should recognise that people within different contexts, groups and subcultures will have different reasons for choosing whether or not to smoke. Future studies should use larger samples with a mixed methods design (quantitative and qualitative).

## Background

Cigarette smoking is the single biggest cause of preventable morbidity and mortality in Australia and worldwide, responsible for 19,000 deaths per annum in 1998 in Australia [1]. Young people have the highest rate of smoking prevalence. In 2004, 17.4% of the Australian population aged 14 years and over reported smoking daily, whereas for the 14-29 year old age group the prevalence was at 34.2% [2].

Numerous researchers studied risk factors associated with smoking uptake, such as lower socioeconomic status and young age, however, few have sought to examine the various protective factors associated with smoking resilience. The majority of resilience research is limited to investigations of single variables and their relationship to resilience. The current exploratory study has made an attempt to bring together variables identified as promoting resilience, and to examine the strength of the relationships.

Positive psychologists define resilience as the interplay between personal characteristics and environmental factors resulting in the ability to overcome adversity [3]. Antonovsky's salutogenic model explores factors assisting individuals in being resilient despite exposure to major life stressors. Rather than seeking the causes of disease, Antonovsky recommended focusing on the causes of successful and positive individual health [4]. Resilience, or resistance to adverse outcomes, has been associated with protective factors both within and outside the individual. Factors within the individual are: sense of coherence (an important internal resource assisting individuals in dealing with stress that is applicable to all cultures and demographic contexts) [5], sense of humour, positive coping style, and mental health. External factors in the social environment are: family, peers, and community.

In particular, sense of humour is recognised as a buffer that improves the capacity to cope and experience positive feelings, despite adversity [6-11]. It can promote resilience within both interpersonal and intrapersonal contexts. It is therefore plausible that those who do not use humour are less resilient and more likely to smoke and suffer from tobacco related illnesses.

Research also shows a strong association between coping style and smoking status. A study comparing smokers with those who have never smoked found that the latter group used active problem solving to cope with stress (confrontative coping) whereas smokers used avoidant coping such as distraction and substance abuse [12].

People without mental illness are also more likely to exhibit smoking resilience. South Australian data showed that 39.4% of individuals with a self-reported mental illness smoked, compared to 22.7% for people with no mental illness [13]. In contrast with the progressive decline of tobacco smoking rates in the general population, smoking rates for individuals with depression continue to increase [14]. As with depression, low levels of anxiety were also associated with smoking resilience. Johnson, et al. [15] tested this association in a sample of late adolescents and young adults and concluded that smoking could moderately predict the development of anxiety disorders. Subsequent research confirmed these results [16,17]. Stress, a milder form of anxiety, can also contribute to smoking initiation [18,19]. Those who successfully manage stress and quickly recover from stressful situations have increased smoking resilience [20].

Moreover, it is widely accepted that family support and in particular parental support, is highly important in building resilience in young people [21]. Young peers are another strong influence. Krosnick and Judd [22] suggested that parental influence does not decrease in adolescence, but rather, the influence of peers increases. Peers can both promote and deter the use of tobacco [23].

In addition to positive social support from family and peers, resilience in young people can also be influenced by community support. The emphasis on community support is consistent with resilience research stressing the importance of the socioecological context in helping young people to avoid the negative effects of risk factors [21].

The current study chose to explore the problem of resilience in the context of smoking among young people. In particular, the study focused on young people of lower socioeconomic status who, despite the odds, do not smoke. The vocational education students from TAFE (Technical and Further Education) have particularly high smoking rates, 44.6% [24] compared with the average of 21.9% in the adult population of 15 years and over [25]. For this reason and due to the fact that many TAFE students come from low socioeconomic backgrounds, this group was targeted for the current study. The main aim of this study was to identify factors contributing to smoking resilience among young people at risk.

## Methods

### Participants and Setting

A total of 92 questionnaires were handed out, and 92 completed questionnaires were returned (100% response rate). The sample consisted of 37 male and 55 female students. The majority did not smoke and were of lower socioeconomic status. Table 1 presents a summary of selected demographic statistics for the sample. Participants were students from the TAFE Adelaide College of the Arts campus (vocational education) from classes across all year levels (year 1, 2 and 3) in Bachelor of Dance Performance, Advanced Diploma of Arts, and Bachelor of Visual Arts and Applied Design. Eligibility was limited to young people, defined as 15-29 years of age. Compared with other TAFE campuses in the metropolitan region, the Adelaide College of the Arts had the highest rate of students who had or were eligible for concession fees, and who were thus classified as being of lower socioeconomic status. This campus was therefore chosen given the current study's focus on researching resilience in young people of lower socioeconomic status, i.e. resilient despite the odds. Eligibility for concession fees required that students or their caregivers held a valid concession card, for example, the Health Care Card. Eligibility is assessed by Centrelink (an Australian commonwealth department supporting people in financial need) who assess the socioeconomic status of applicants [26]. The survey asked students to indicate whether they were on or eligible for concession. Given that the majority of students (79.3%) were on concession, the sample was representative of a campus regarded as being of lower socioeconomic status.

**Table 1:** Demographic Summary of Student Characteristics

Demographic Characteristics		*n (%)*
**Gender**	Male	37 (40.0)
	Female	55 (60.0)
**Age in years**		
	15-17	2 (5.40)
	18-20	20 (54.05)
	21-23	9 (24.32)
	24-26	4 (10.81)
	27-29	2 (5.40)
		
**Lower Socioeconomic Status**	Yes	73 (79.3)
	No	19 (20.7)
**Smoking Status**	Yes	25 (27.0)
	No	67 (73.0)

### Course Type and Smoking Status

**Figure 1:**
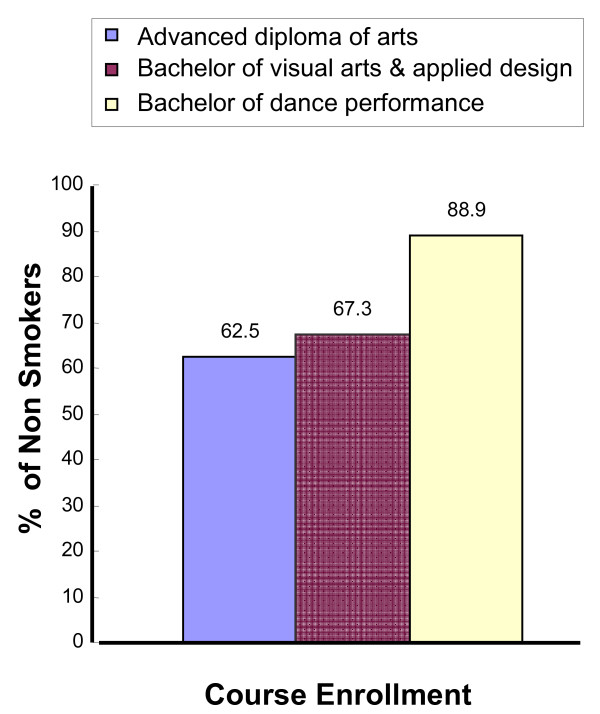


Of the 92 students, 17.39% (n = 16) were enrolled in the Advanced Diploma of Arts (Diploma of Arts), 53.26% (*n *= 49) in the Bachelor of Visual Arts and Applied Design (Visual Arts), and 29.35% (*n *= 27) in the Bachelor of Dance Performance (Dance). Of the students in Diploma of Arts, 37.5% (*n *= 6) were smokers, and 62.5% (*n *= 10) were non smokers. For Visual Arts, 32.7% (*n *= 16) were smokers, and 67.3% (*n *= 33) were non smokers, and in Dance 11.1% (*n *= 3) were smokers, and 88.9% (*n *= 24) were non smokers (See Figure 1). The above descriptive statistics indicate that Bachelor of Dance Performance students have a tendency to not smoke. Of the Dance students, 26 out of 27 were male. Dance students tended not to smoke.

### Materials

Participants received a nine page self-report questionnaire consisting of 101 questions (see Additional file 1, Questionnaire) divided into sections on sense of humour, style of coping, social support (family, peers, and community), sense of coherence, and depression, anxiety and stress (DASS). Background information included questions on smoking behaviour, for example, whether or not participants smoked, parental approval and disapproval, course type, and students' reported reasons for not smoking. Due to low numbers of daily smokers, participants were labelled as smokers if they smoked daily, weekly, or monthly. All scales except DASS asked participants to answer questions based on the past month, and DASS for the past week.

#### Sense of Coherence Scale

Sense of coherence (SOC) was based on Antonovsky's [27] Orientation to Life Questionnaire, more commonly known as the 'Sense of Coherence Scale'. The shortened 13 item version was used. Five items were reverse scored. Wording was slightly modified for the sample. Total range of possible scores were 13-65, with higher scores (45-65) indicating higher SOC. The SOC scale has been widely used and validated [e.g. [18,5,28]]. In 124 studies using the SOC-29, the Cronbach's alpha ranged from 0.70 to 0.95, and in 127 studies using the SOC-13 it ranged from 0.70 to 0.92, supporting claims of validity. The scale is adaptable to all demographics. The Cronbach's alpha in the present study was 0.65.

#### Sense of Humour Scale

The scale for sense of humour was constructed by the researcher for this study. A 5-point rating scale was used, ranging from 1 for 'never' to 5 for 'always.' Higher scores (3 to 5) indicated a higher sense of humour. The scale contains three items, two addressing the individuals' own perceptions of their sense of humour, and one regarding other people's perceptions. The latter was included to increase the scale's validity. To test for internal consistency, the Cronbach's alpha for this scale was calculated, showing 0.68.

#### Coping Style for Adults

The scale for coping style was based on the 'Coping Style for Adults' (CSA) by Frydenberg and Lewis [29], and was modified for consistency with Holahan and Moos' [30] theory of avoidant and confrontative coping. Two items measured avoidant coping and two measured confrontative coping. The wording was slightly modified for a younger population. A 5-point rating scale ranged from 1 for 'never' to 5 for 'always'. Moderate to high scores were represented by responses of 3 to 5 on individual items. High scores for questions 1 ("How often did you try to cope with problems by talking about them to other people?") and 3 ("How often did you reflect on problems, plan solutions, and tackle problems systematically?") indicated a moderate to high confrontative coping style, whereas high scores for questions 2 ("How often did you try to cope with problems by keeping them to yourself?") and 4 ("How often did you consciously block out problems?") indicated a moderate to high avoidant coping style. Frydenberg and Lewis [29] claimed validity for the CSA scale based on analysis of five studies reporting significant relationships between desired outcomes (stress-free) and productive coping strategies. Similarly, Holahan and Moos [30] enjoy widespread support and acceptance for their theory on avoidant and confrontative coping. The Cronbach's alpha for the current study's modified scale was 0.62.

#### Depression, Anxiety and Stress Scale (DASS)

Depression, anxiety and stress were measured using the 42 item 'Depression, Anxiety, and Stress Scale' (DASS) developed by Lovibond and Lovibond [31]. The scale ranged from 0 for 'did not apply to me at all,' to 3 for 'applied to me very much or most of the time.' Higher scores indicated higher levels of depression, anxiety and stress. Moderate to high levels of depression were represented by 14-28+, for anxiety 10-20+, and stress 19-34+. The DASS is widely used with strong claims of validity [e.g. [32,31]]. In particular, Lovibond and Lovibond's [31] support for the validity of the scale is based on a study using predominantly students, thereby arguably increasing validity for subsequent studies also using students. The Cronbach's alpha for this scale was 0.96. The scale can be used with participants as young as twelve.

#### Multidimensional Support Scale

The scale on social support for family, peers and community was based on the 'Multidimensional Support Scale' (MDSS) developed by Winefield, Winefield and Tiggeman [33]. Minor modifications were made, for example, the Likert scale was reduced from a 7-point to a 5-point scale for consistency, ranging from 1 for 'never' to 5 for 'always.' Family and peer support measures consisted of six items each, and three for community support. Higher scores indicated higher support levels. Validity of the MDSS was addressed by Winefield et al. [33] by conducting multiple linear regressions, showing that support measures significantly increased the amount of explained variance in well-being measures. The authors removed one item from the questionnaire due to poor validity (How often do you tell jokes and chatter?). This item was not included in the current study. The MDSS is flexible and adaptable for use with different populations. The Cronbach's alpha for the current study's modified scale was 0.87 for family and peers, and 0.91 for community.

The additional factors: parental approval and disapproval, course type, and students' reasons for not smoking, were researched using information obtained from the Background Information section of the survey. Parental approval and disapproval was measured by asking students to tick a box (either 'approves' or 'disapproves') for the following questions: "How does your mother/female caregiver feel about smoking" and "How does your father/male caregiver feel about smoking." Information on course type was obtained by asking for the full name of the course the students were studying. Students' reasons for not smoking were measured by asking, "If you haven't taken up smoking (daily) why?" They were asked to tick one box, choosing the main reason from the following options: influence of friends, influence of family, influence of people in the community, health or fitness reasons, it's not cool/people would look down on me, other.

### Procedure

Questionnaires were handed to students during class. The teacher notified students at the start of class that a research student would arrive towards the end of class inviting voluntary participation in a research study. The researcher commenced with a brief presentation on the purpose and nature of the study, including information on voluntary participation and management of privacy and confidentiality. Data were collected over a two week period in six classes. The researcher chose to present the questionnaires personally in order to answer any questions, and to maximise interest and subsequent response rate.

### Analysis

Categorical variables are presented as frequencies and percentages. Continuous variables are presented as means and standard deviations. Independent sample t-tests (two-sided) were conducted to compare smokers and non-smokers with respect to normally distributed continuous variables of interest. To examine the extent to which the predictor variables were able to classify people as smokers or non-smokers, a logistic regression was conducted using 'do you currently smoke' as the dependent variable (recoded 0 = non-smoker, 1 = smoker) with peer support levels as the predictor. An exploratory analysis comparing depression, anxiety and stress scores in relation to how often a person smoked (i.e. daily, weekly, or monthly) was conducted with one-way analysis of variance (ANOVA). In order to determine which groups differed from each other we used the Tukey's honestly significant difference (HSD) test. Pearson's correlation was performed to observe relationships between parental approval/disapproval and smoking status.

### Ethics

Approval for the study was given by the University of Adelaide Human Research Ethics Committee.

## Results

### Smoking Status and Psychological and Social Variables

No significant differences were found for smoking status in relation to sense of coherence, sense of humour, coping style, family support and community support. A relationship was found between peers and smoking status (Table 2). Additional exploratory analyses compared depression, anxiety and stress scores in relation to how often a person smoked (daily, weekly or monthly). A summary of these findings, including the results for a one-way ANOVA is provided in Table 3. Post-hoc tests (Tukey's HSD) showed that scores for all three measures were significantly higher in the daily smoking group than in the other two groups. However, when all smokers and all non-smokers were compared on depression, anxiety and stress no difference was detected (p > .05).

**Table 2:** Predictor of smoking status

Variable	β	Wald	Odds Ratio
**Peer Social Support**	.77	4.13 *	2.15 *

* *p *< .05

**Table 3:** Levels of Depression, Anxiety, and Stress according to Smoking Frequency (Daily, Weekly, Monthly)

	Daily *M (SD)*	Weekly *M (SD)*	Monthly *M (SD)*	*F *(2, 22)
**Depression**	15.50 (4.20)	5.50 (4.20)	5.08 (6.29)	3.58 *
**Anxiety**	14.62 (12.58)	5.25 (3.40)	5.46 (4.20)	3.75 *
**Stress**	19.75 (11.02)	8.25 (7.23)	8.46 (8.06)	4.31 *

* *p *< .05

### Peer Support and Smoking Status

Smokers reported significantly higher peer support (*M *= 4.00, *SD *= .54) than non smokers (*M *= 3.67, *SD *= .71), *t *(90) = 2.10, *p *< .05 (Cohen's *d *= .44). The Cohen's d indicates a moderate effect size.

A logistic regression analysis (Table 2) showed that, for each unit increase in peer support scores, a student was 2.15 times more likely to be a smoker. The percentage of cases correctly classified was 73% indicating that the model was substantially better than chance in its ability to classify cases.

### Parental Approval/Disapproval, and Smoking Status

Smoking status in relation to parental approval and disapproval was examined to determine whether parents potentially influenced their children's smoking status. No young people reported smoking if their fathers approved and mothers disapproved of their smoking (0 out of 10) and only 2 out of 12 smoked when their fathers disapproved and their mother approved. When analyses were confined only to those who smoked, a strong correlation was found between parental approval (both parents) and the frequency of smoking (coded as separate categorical variables in point bi-serial correlations). If there was parental approval, then students tended to be less frequent smokers: (*r *(22) = -.90, *p *< .01).

### Family Support and Smoking Status

A very strong positive correlation was found between weekly smoking, and the item, 'family really tried to listen and understand' (*r *(22) = .95, *p *< .05). A very strong positive correlation was also found for weekly smoking and the item 'using family as an example of solving problems' (*r *(79) = .95 *p *< .05)

### Smoking Status according to Parental Approval/Disapproval and Gender

Gender differences were also considered in relation to the relationship between smoking status and parental approval. For males whose parents did not approve, there were 16 non smokers and 10 smokers, whereas for females, there were 34 non smokers, 9 smokers if parents disapproved. There was no significant relationship between gender and smoking status in this subsample (p > .05) as indicated by a chi-squared test, but there was a trend toward a relatively higher number of smokers in the male group.

### Students' Reported Reasons for Not Smoking

Table 4 presents the reasons given by students for not smoking. Health and fitness were reported the most frequent reasons these students did not smoke.

**Table 4:** Frequency scores for Reasons Given for Not Smoking

Reasons for Not Smoking	*n (%)*
**Health or Fitness Reasons**	41 (49.4)
**Other**	21 (25.3)
**Influence of Family**	10 (12.0)
**Influence of Friends**	6 (7.2)
**Influence of people in the Community**	3 (3.6)
**It's not cool, people will look down on me**	2 (2.4)
**Total**	83 (100.0)

## Discussion

The aims of the current exploratory study were to bring together a number of variables related to resilience and smoking status and study them in the one investigation. The study sought to identify factors contributing to smoking resilience among young people at risk, i.e. those who despite the odds do not smoke.

Previous studies have indicated relationships between smoking status and the following factors: sense of coherence, sense of humour, coping style, depression, anxiety, stress, and social support (family, peers, and community). The present study found a relationship between peers and smoking status (smoking or non smoking), but other expected differences were not obtained. Other analyses revealed a relationship between smoking status with smoking frequency, parental approval, course type, and health and fitness.

Although no association was found between smokers and non smokers for depression, anxiety and stress, an association was found for frequency of smoking (daily, weekly, monthly). Scores for depression, anxiety and stress were higher for daily smokers. This is consistent with previous studies that reported depression, anxiety and stress using daily measures for smoking [34]. Given the limited research on smoking frequency, it would be useful to replicate the current study's measures for daily, weekly and monthly smoking. In addition, questions regarding the type and strength of cigarettes smoked could provide useful insights.

The current study found an association between family support and smoking status. Very strong positive correlations were found between weekly smoking and the items 'how often did your family really try to listen when you talked about your problems or worries?' and 'how often could you use them as examples of how to deal with problems or worries?' This indicated that students who smoked weekly perceived their family as trying to listen to them more, and more frequently regarded them as examples of how to deal with problems. Progression to weekly smoking may feasibly have captured the attention and concern of parents or other family members (whether they directly noticed the smoking or other accompanying changes in behaviour), which could explain the results. However, previous research reported that daily smokers tended to believe that the adults who were most significant to them would most likely approve of them smoking [35]. Perhaps these smokers had conflicting feelings about their smoking status, and in an effort to reduce the discomfort (cognitive dissonance) they decided that others approved of their smoking. Differences between daily, weekly, and monthly smoking warrant further attention as this may inform smoking assessment and prevention programmes.

Exploratory analyses showed that students in the Dance course had a tendency to not smoke. Smoking is an incompatible choice given that exercise is an integral and ongoing component of the course. Braverman's [36] study of social status suggested that boys avoided smoking and gained social status through participation in sport. As with Braverman's study, young people in the Dance course were also predominantly exposed to non smokers, and therefore in regards to smoking resilience they were associating with peers of positive influence. This is an important finding. It shows that given the right motivation, young people will avoid smoking uptake. A literature review showed that overall, young people did not respond to messages about the health consequences of smoking if their motivation for smoking was stronger and more overpowering, such as fulfilling a need for social intimacy or lubricating social interactions [37,38]. Furthermore, given that most students in Dance were male (26 out of 27), future research could explore these gender differences.

Consistent with this, the majority of students chose 'health and fitness' as the reason for not smoking. This provides an opportunity for future research to expand on health and fitness as a motivating factor to not smoke. A number of students also ticked 'other' among the options presented. Future research could ideally measure this item using a qualitative design.

The majority of research has tried to predict smoking but very little has been done on issues of resilience despite the odds. Given that resilience can be taught, an understanding of smoking resilience predictors can have implications for interventions. Single anti-smoking messages cannot be generalised to all young people, but should recognise that different contexts, groups and subcultures will have different reasons for choosing whether or not to smoke. The findings also highlight the importance of utilising a positive approach in understanding human behaviour. For example, young people with a commitment to and enjoyment of Dance can view this activity as a strength, which might not only contribute to smoking resilience but could also be transferred to numerous other domains such as resilience against bullying, and moral resilience. This supports results of previous studies that emphasised the importance of individual strengths and virtues as valuable resources [27,39,40]. If viewed from a socioecological perspective, the findings illustrate the interplay between the individual and the social environment. Therefore, by creating supportive environments specific to the needs of a particular group type (such as the Dance students) healthy non smoking lifestyles may be successfully promoted.

The design of the study is limited by inherent problems of self report measures. The validity of self-reported smoking in particular is often questioned due to the belief that smokers tend to deny smoking [41]. This could account for the lack of significant differences found between smokers and non smokers. In addition, due to the low number of reported daily smokers, the scores for weekly and monthly smokers were added to form a combined score. A larger sample would yield higher daily smokers, thereby increasing the power of comparison across all factors. Results are limited to the sample of TAFE students and cannot be generalised to the population. However, the study highlights the importance of recognising that different individuals within different settings will yield unique results for smoking resilience.

Future studies should also use a mixed methods design (quantitative and qualitative) and could be longitudinal, ideally enlisting students at commencement of their course through to completion to identify factors that affect smoking resilience.

## Conclusions

Young people with peer support tended to smoke. A relationship between daily smoking and depression, anxiety and stress was also found. When both mothers and fathers disapproved of their children smoking, then fewer children smoked, but this influence was greater on females compared with males. The majority of students chose 'health and fitness' as a reason for not smoking. Single anti-smoking messages cannot be generalised to all young people, but should recognise that people within different contexts, groups and subcultures will have different reasons for choosing whether or not to smoke. Future studies should use larger samples with a mixed methods design (quantitative and qualitative).

## Competing interests

The authors declare that they have no competing interests.

## Authors' contributions

YC contributed to the design of this study, conducted an exploratory survey of the literature, recruited participants, conducted the survey and analysis, and co-drafted this paper. DT contributed to the design of this study, interpretation of results and supervised the overall project. AMW contributed to the design of this study, interpretation of results and co-drafted this paper. PD contributed to the design of this study and its analysis, as well as interpretation of results. All authors read and approved the final manuscript.

## Supplementary Material

Additional file 1 **Questionnaire**. File 1 contains the questionnaire, which includes Background Information and the Depression, Anxiety and Stress Scale (DASS). Click here for file
