# Supplementary material for: Determinants of resilience to cigarette smoking among young Australians at risk: an exploratory study
Source: Tob Induc Dis. 2010 Jul 8;8(1):7. doi: 10.1186/1617-9625-8-7 (PMC2914043; doi:10.1186/1617-9625-8-7)
Supplement: Additional file 1 — Questionnaire. File 1 contains the questionnaire, which includes Background Information and the Depression, Anxiety and Stress Scale (DASS). [file 1617-9625-8-7-S1.PDF]

Appendix A

Questionnaire

Questionnaire

Here are a series of questions relating to various aspects of life. The first set of questions ask about your **sense of humour** IN THE LAST MONTH. For each question and statement please indicate the extent to which you agree or disagree by putting a circle around the appropriate number. There are no right or wrong answers.

	Never	Almost never	Sometimes	Often	Always
How often would you say you used your sense of humour?	1	2	3	4	5
How often would you say others thought of you as having a sense of humour?	1	2	3	4	5
How often did you use humour to help you cope with your problems or worries?	1	2	3	4	5

The next set of questions are about **style of coping.** Think about problems you've had in THE LAST MONTH.

	Never	Almost Never	Sometimes	Often	Always
How often did you try to cope with problems by talking about them to other people?	1	2	3	4	5
How often did you try to cope with problems by keeping them to yourself?	1	2	3	4	5
How often did you reflect on problems, plan solutions, and tackle problems systematically?	1	2	3	4	5
How often did you consciously block out problems?	1	2	3	4	5

The following questions refer to your current **Social Supports** (Family, Friends, & Community) that were available to you IN THE LAST MONTH. Firstly, think of your **Family**, especially the 2-3 people who are most important to you. For each question, please circle your answer.

	Never	Almost Never	Sometimes	Often	Always
How often did your **family** really try to listen when you talked about your problems or worries?	1	2	3	4	5
How often did you feel that they were really trying to understand your problems or worries?	1	2	3	4	5
How often did they make you feel loved?	1	2	3	4	5
How often did they offer to help you in practical ways, like doing things for you or lending you money?	1	2	3	4	5
How often did they answer your questions or offer you advice about your problems or worries?	1	2	3	4	5
How often could you use them as examples of how to deal with problems or worries?	1	2	3	4	5

Now think of your **peers** (friends) IN THE LAST MONTH, especially the 2-3 who are most important to you.

	Never	Almost Never	Sometimes	Often	Always
How often did your **peers** really try to listen when you talked about your problems or worries?	1	2	3	4	5
How often did you feel that they were really trying to understand your problems or worries?	1	2	3	4	5
How often did they make you feel loved?	1	2	3	4	5
How often did they offer to help you in practical ways, like doing things for you or lending you money?	1	2	3	4	5
How often did they answer your questions or offer you advice about your problems or worries?	1	2	3	4	5
How often could you use them as examples of how to deal with problems or worries?	1	2	3	4	5

Now think of your **community** supports, such as recreation and sporting clubs, the gym, church & other religious groups, cultural groups, workplace, schools, community organisations etc. Think of those people in the community that have been most important to you in THE LAST MONTH

Question	Never	Almost Never	Sometimes	Often	Always
How often were you involved in community groups?	1	2	3	4	5
How often did you feel a strong sense of welcome from your community groups?	1	2	3	4	5
How often did you feel that your community groups were supportive of you and improved your quality of life?	1	2	3	4	5

The next set of questions ask about some general feelings you might have about yourself and other people (**sense of coherence**) IN THE LAST MONTH

Question	Never	Almost Never	Sometimes	Often	Always
How often do you have the feeling that you don't really care about what goes on around you?	1	2	3	4	5
How often has it happened that you were surprised by the behaviour of people who you thought you knew well?	1	2	3	4	5
Has it happened that people who you counted on disappointed you?	1	2	3	4	5
How often have you felt that your life has had very clear goals and purpose?	1	2	3	4	5
How often have you had the feeling that you were being treated unfairly?	1	2	3	4	5
How often did you have the feeling that you were in an unfamiliar situation and didn't know what to do?	1	2	3	4	5
How often did you get deep pleasure and satisfaction from doing the things you do every day?	1	2	3	4	5
How often did you have very mixed-up feelings and ideas?	1	2	3	4	5
How often did you have feelings inside that you would rather not feel?	1	2	3	4	5

	Never	Almost Never	Sometimes	Often	Always
Many people, even those with a strong character, sometimes feel like 'losers' in certain situations. How often did you feel this way?	1	2	3	4	5
When something happened, how often did you find that you generally overestimated or underestimated its importance?	1	2	3	4	5
How often did you have the feeling that there's little meaning in the things you do in your daily life?	1	2	3	4	5
How often did you have feelings that you weren't sure you could keep under control?	1	2	3	4	5

The questions on the following page ask about your general emotional well being (**DASS**)

DASS

Please read each statement and circle a number 0, 1, 2 or 3 which indicates how much the statement applied to you *over the PAST WEEK.* There are no right or wrong answers. Do not spend too much time on any statement.

The rating scale is as follows:

0 Did not apply to me at all
1 Applied to me to some degree, or some of the time
2 Applied to me to a considerable degree, or a good part of time
3 Applied to me very much, or most of the time

1	I found myself getting upset by quite trivial things	0	1	2	3
2	I was aware of dryness of my mouth	0	1	2	3
3	I couldn't seem to experience any positive feeling at all	0	1	2	3
4	I experienced breathing difficulty (eg, excessively rapid breathing, breathlessness in the absence of physical exertion)	0	1	2	3
5	I just couldn't seem to get going	0	1	2	3
6	I tended to over-react to situations	0	1	2	3
7	I had a feeling of shakiness (eg, legs going to give way)	0	1	2	3
8	I found it difficult to relax	0	1	2	3
9	I found myself in situations that made me so anxious I was most relieved when they ended	0	1	2	3
10	I felt that I had nothing to look forward to	0	1	2	3
11	I found myself getting upset rather easily	0	1	2	3
12	I felt that I was using a lot of nervous energy	0	1	2	3
13	I felt sad and depressed	0	1	2	3
14	I found myself getting impatient when I was delayed in any way (eg, lifts, traffic lights, being kept waiting)	0	1	2	3
15	I had a feeling of faintness	0	1	2	3
16	I felt that I had lost interest in just about everything	0	1	2	3
17	I felt I wasn't worth much as a person	0	1	2	3
18	I felt that I was rather touchy	0	1	2	3
19	I perspired noticeably (eg, hands sweaty) in the absence of high temperatures or physical exertion	0	1	2	3
20	I felt scared without any good reason	0	1	2	3
21	I felt that life wasn't worthwhile	0	1	2	3

Reminder of rating scale:

0 Did not apply to me at all
1 Applied to me to some degree, or some of the time
2 Applied to me to a considerable degree, or a good part of time
3 Applied to me very much, or most of the time

22	I found it hard to wind down	0	1	2	3
23	I had difficulty in swallowing	0	1	2	3
24	I couldn't seem to get any enjoyment out of the things I did	0	1	2	3
25	I was aware of the action of my heart in the absence of physical exertion (eg, sense of heart rate increase, heart missing a beat)	0	1	2	3
26	I felt down-hearted and blue	0	1	2	3
27	I found that I was very irritable	0	1	2	3
28	I felt I was close to panic	0	1	2	3
29	I found it hard to calm down after something upset me	0	1	2	3
30	I feared that I would be "thrown" by some trivial but unfamiliar task	0	1	2	3
31	I was unable to become enthusiastic about anything	0	1	2	3
32	I found it difficult to tolerate interruptions to what I was doing	0	1	2	3
33	I was in a state of nervous tension	0	1	2	3
34	I felt I was pretty worthless	0	1	2	3
35	I was intolerant of anything that kept me from getting on with what I was doing	0	1	2	3
36	I felt terrified	0	1	2	3
37	I could see nothing in the future to be hopeful about	0	1	2	3
38	I felt that life was meaningless	0	1	2	3
39	I found myself getting agitated	0	1	2	3
40	I was worried about situations in which I might panic and make a fool of myself	0	1	2	3
41	I experienced trembling (eg, in the hands)	0	1	2	3
42	I found it difficult to work up the initiative to do things	0	1	2	3

Background Information

Please tick the box that best describes

1. Age:
   - ❑ 15-17
   - ❑ 18-20
   - ❑ 21-23
   - ❑ 24-26
   - ❑ 27-29
2. Gender:
   - ❑ Male
   - ❑ Female
3. Are you currently studying full time or part time?
   - ❑ Full time student
   - ❑ Part-time student
4. What is the full name of the course you are studying? ……………………………………………………………………………………
5. What year are you in?
   - ❑ Year 1
   - ❑ Year 2
   - ❑ Year 3
6. Are you currently in paid employment?
   - ❑ Full-time employed
   - ❑ Part-time employed
   - ❑ Not applicable
7. Are you currently on/eligible for Concession?
   - ❑ Yes
   - ❑ No
8. In which country were you born?
   - ❑ Australia
   - ❑ New Zealand
   - ❑ UK and Ireland
   - ❑ North America
   - ❑ Other Europe [Please specify..............................]
   - ❑ Other [……………………………………]
9. Do you identify as Aboriginal or Torres Strait Islander?
   - ❑ No
   - ❑ Aboriginal
   - ❑ Torres Strait Islander
   - ❑ Both

10. What is the main language spoken at home?

- ❑ English
- ❑ Another language [please specify…………………………………]

11. In which council area do you live?

- ❑ Charles Sturt Council
- ❑ Port Adelaide & Enfield
- ❑ Adelaide City
- ❑ Prospect
- ❑ West Torrens
- ❑ Walkerville
- ❑ Other………………………………[If not sure of your council area, please write postcode ……….………]

12. Before you came to TAFE what was the highest level of Qualification received?

- ❑ Up to Year 10
- ❑ Up to Year 11
- ❑ Up to Year 12
- ❑ Previous TAFE / Diploma
- ❑ University Degree / Tertiary education

13. Do you currently smoke cigarettes?

- ❑ Daily [How many per day?........................]
- ❑ At least weekly (i.e. not every day) [How many per week?.....................]
- ❑ Less often than weekly (i.e. not every week) [How many per month?..............]
- ❑ Not at all

14. In the past, have you ever been a daily smoker for more than 1 month?

- ❑ Yes
- ❑ No

15. If you have tried smoking, about what age were you when you smoked your first cigarette? ………………….

16. If you currently smoke (daily) why did you take up smoking? [please tick one box. Choose the main reason]

- ❑ Influence of Friends
- ❑ Influence of Family
- ❑ Influence of people in the community
- ❑ I felt depressed and/or anxious
- ❑ I had problems/worries
- ❑ To be cool / have people look up to me
- ❑ Other

17. If you haven’t taken up smoking (daily) why? [please tick one box. Choose the main reason]

- ❑ Influence of Friends
- ❑ Influence of Family
- ❑ Influence of people in the community
- ❑ Health or fitness reasons
- ❑ It’s not cool / people would look down on me
- ❑ Other

18. If you haven’t tried smoking, do you plan to in the future?

- ❑ Yes
- ❑ No
- ❑ Don’t know

19. Other than yourself, are there other members of the household who are smokers?

[Tick as many as apply]

- ❑ No
- ❑ Brothers or Sisters
- ❑ Partner (boy/girlfriend, defacto partner, or husband/wife)
- ❑ Parent
- ❑ Mother / stepmother / adopted mother / female caregiver
- ❑ Father / stepfather / adopted father / male caregiver

20. How does your mother /female caregiver feel about smoking?

- ❑ Approves
- ❑ Doesn’t approve

21. How does your father/male caregiver feel about smoking?

- ❑ Approves
- ❑ Disapproves

22. Do your very best/closest friends smoke?

- ❑ Yes
- ❑ No

23. Do most of your friends smoke?

- ❑ Yes
- ❑ No

24. How do most of your friends feel about smoking?

- ❑ Approve
- ❑ Disapprove

Thank you for taking the time to complete the questionnaire!
